# Simultaneous fluorescence imaging of bowel perfusion and ureter delineation using methylene blue: a demonstration in a porcine model

**DOI:** 10.1007/s00464-023-10142-6

**Published:** 2023-05-30

**Authors:** Danique J. I. Heuvelings, Zaid Al-Difaie, Max H. M. C. Scheepers, Nariaki Okamoto, Michele Diana, Laurents P. S. Stassen, Nicole D. Bouvy, Mahdi Al-Taher

**Affiliations:** 1 NUTRIM School of Nutrition and Translational Research in Metabolism, Maastricht University, P.O. Box 5800, 6202 AZ Maastricht, The Netherlands; 2 GROW School for Oncology and Developmental Biology, Maastricht University, Maastricht, The Netherlands; 3 IRCAD, Research Institute Against Digestive Cancer, Strasbourg, France; 4 ICube Laboratory, Photonics Instrumentation for Health, Strasbourg, France; 5 Department of Digestive and Endocrine Surgery, University Hospital of Strasbourg, Strasbourg, France; 6 Department of Surgery, Maastricht University Medical Center, Maastricht, The Netherlands

**Keywords:** Perfusion assessment, Ureteral delineation, Methylene blue, Indocyanine green, Intraoperative near-infrared fluorescence imaging, Anastomotic leakage

## Abstract

**Background:**

Intraoperative near-infrared fluorescence imaging (NIRF) with preoperative optical dye administration is a promising technique for quick and easy intraoperative visualization of the ureter and for an improved, real-time assessment of intestinal perfusion. During colorectal surgery, there is a need for simultaneous non-invasive ureteral imaging and bowel perfusion assessment, using one single camera system. The purpose of this study is to investigate the feasibility of simultaneous intestinal perfusion and ureteral imaging using a single commercially available NIRF imaging system.

**Methods:**

Six Landrace pigs underwent laparotomy under general anesthesia in this experiment. An intravenous (IV) dose of 0.2 mg/kg indocyanine green (ICG) was given to assess bowel perfusion. Two pairs received a methylene blue (MB) iv injection of 0.75, 0.50 or 0.25 mg/kg respectively to investigate ureteral visualization. Quest Spectrum Fluorescence Camera (Quest Medical Imaging, Middenmeer, The Netherlands) was used for NIRF imaging.

**Results:**

Ureter visualization and bowel perfusion under NIRF imaging was achieved in all animals. All ureters were visible after five to ten minutes and remained clearly visible until the end of every experiment (120–420 min). A mixed model analysis did not show any significant differences neither between the three groups nor over time. Importantly, we demonstrated that bowel perfusion could be visualized with methylene blue (MB) as well. We observed no interference between ICG and MB and a faster washout of MB.

**Conclusion:**

We successfully demonstrated simultaneous fluorescence angiography with ICG and ureteral imaging with MB in the same surgical procedure, with the same commercially available NIRF imaging equipment. More importantly, we showed that the use MB is adequate for bowel perfusion assessment and ureter visualization with this NIRF imaging system. Besides, MB showed an earlier washout time, which can be clinical beneficial as a repeated dye injection may be necessary during a surgical procedure.

**Supplementary Information:**

The online version contains supplementary material available at 10.1007/s00464-023-10142-6.

## Introduction

Anastomotic leakage (AL) is one of the most dreaded complications after colorectal surgery. Probably the most important cause of AL is impaired perfusion of the bowel. Assessment of bowel perfusion is therefore one of the crucial strategies in reducing the incidence of AL [1, 2]. Another feared complication during colorectal surgery is ureteral injury. In order to prevent iatrogenic damage, the surgeon must be aware of the exact location of the ureter.

Intraoperative near-infrared fluorescence imaging (NIRF) with preoperative optical dye administration is a technique for quick and easy intraoperative visualization of the ureter [3–5] and for an improved assessment of anastomotic perfusion [2, 6–11]. However, to date there is no clinical study which evaluates simultaneous fluorescence-enhanced ureteral delineation and intestinal perfusion in the same surgical procedure over time and the possibility of using one single dye.

Over the last decade, (pre-)clinical studies have been performed to visualize the ureter. Due to the exclusive clearance of indocyanine green (ICG) by the liver, it is not suitable for ureteral imaging since it is not cleared in the urine. Methylene blue (MB) on the other hand, a clinically approved and widely used dye, is excreted by the kidneys and can consequently be administered for non-invasive ureteral imaging. However, results of clinical and pre-clinical experiments investigating the feasibility of MB for ureteral imaging have shown conflicting results regarding its added clinical value [3, 4]. This may be due to the characteristics of the dye itself, having only a weak fluorescent signal, or to the laparoscopic equipment used. The latter refers to a disadvantage of MB, which is excited at ~ 670 nm, in contrast to other dyes such as ICG which is excited at ~ 800 nm. As a result, the use of MB requires specifically developed equipment. The vast majority of imaging systems used in the studies with MB thus far were experimental and not commercially available for clinical use [5].

In colorectal surgery, there is a need for simultaneous non-invasive ureteral imaging and bowel perfusion assessment. The latter can be achieved by finding a single dye that can simultaneously identify these structures, or an adequate NIRF imaging system that can simultaneously identify these structures with two different dyes. Our group has already successfully studied and reported on the first approach [12]. However, this was a pre-clinical study that is not yet ready for clinical implementation. The use of two dyes simultaneously for ureteral imaging and bowel perfusion imaging has become potentially feasible now that a commercial imaging system is available for such an approach.

The aim of this study was to investigate the feasibility of simultaneous intestinal perfusion and ureteral imaging using a single commercially available NIRF imaging system.

## Materials and methods

This feasibility study was performed at the central animal facilities of Maastricht University (Maastricht, The Netherlands). Animals were used in compliance with Dutch regulations and legislation concerning animal research, and the study was performed according to a protocol approved by the Experimental Animal Committee of Maastricht University (DEC-UM) (approval number: 2017-021-001). Informed written consent was not applicable.

### Animals

A total of six mature (35–45 kg) female Landrace pigs were used for this study. A pig model was chosen because of the anatomical similarities between humans and pigs, and previous successful application of NIRF imaging in pigs [13]. Animals were used in compliance with the regulations of Dutch legislation concerning animal research, and the study was performed according to an approved protocol by the local animal ethics committee.

### Preparation of the dyes

MB (Proveblue, Provepharm Life Solutions, Marseille, France) was diluted in a sterile phosphate-buffered saline (PBS) solution to a concentration of 1 mg/ml. In a previous review, MB doses ranging from 0.25 to 1 mg/kg were studied [14]. A dose of 0.75 mg/kg resulted in the highest target-to-background ratio of the fluorescence image of the ureter [14]. However, in another review by van Manen et al. [15], a dose of 0.25 mg/kg was recommended. In our own experience in a clinical pilot study using this dye, we found that NIRF imaging was strongly influenced by the dose/concentration of the dye [4]. Consequently, in this study, we investigated three different MB doses: two pigs received a bolus IV injection of 0.75 mg/kg (group 1), two other pigs of 0.50 mg/kg (group 2), and the final two pigs 0.25 mg/kg (group 3). Additionally, ICG (Verdye, Diagnostic Green GmbH, Aschheim, Germany) was diluted in a sterile H_2_O solution to a concentration of 2.5 mg/ml. A dose of 0.2 mg/kg was given as a bolus IV injection. This dose is based on the current frequently clinically used dose range in patients as was previously found in our analysis of 1,240 patients registered in the EURO-FIGS registry on fluorescence angiography [16].

### Fluorescence imaging system

The commercially available Quest Spectrum Fluorescence Camera (QUEST SPECTRUM®, Quest Medical Imaging, Middenmeer, The Netherlands) was used for NIRF imaging. To ensure standardized measurements and prevent potential movement of the camera, the camera was fixed with a custom-made mechanic, articulated arm, which was connected to the surgical table. The distance of the camera tip to the target organ was measured with a sterile paper ruler and was 15 cm in all procedures. During NIRF imaging, environmental lights were dimmed preventing ambient light interference. Because of this standardization, and prevention of motion of the camera and animal, we ensured to have high quality of images through the surgical procedure. The 800 nm channel was used to capture ICG fluorescence (ICG mode) while the 700 nm channel was used for MB fluorescence imaging (MB mode). The information captured during recording was visualized within several different fluorescence formats and displayed onto a screen while performing the procedures. First, a color image of the surgical field was presented together with the NIRF image to allow surgical guidance. Additionally, two overlay modes, including a fluorescence intensity map and a NIRF image that is projected over the colored image, were projected onto the same screen.

### Anesthesia

All surgical procedures were performed under general anesthesia. Standard medication used to ensure proper sedation and analgesia was as follows: intramuscular injection of azaperone 3 mg/kg, ketamine 10 mg/kg, atropine 0.05 mg/kg, thiopental 10–15 mg/kg, isoflurane (dose depending on the effect), and oxygen 20–40 ml/kg/min. All alterations in vital parameters were monitored by the animal anesthesiologist.

### Surgical procedure and measurements

**Fig. 1:**
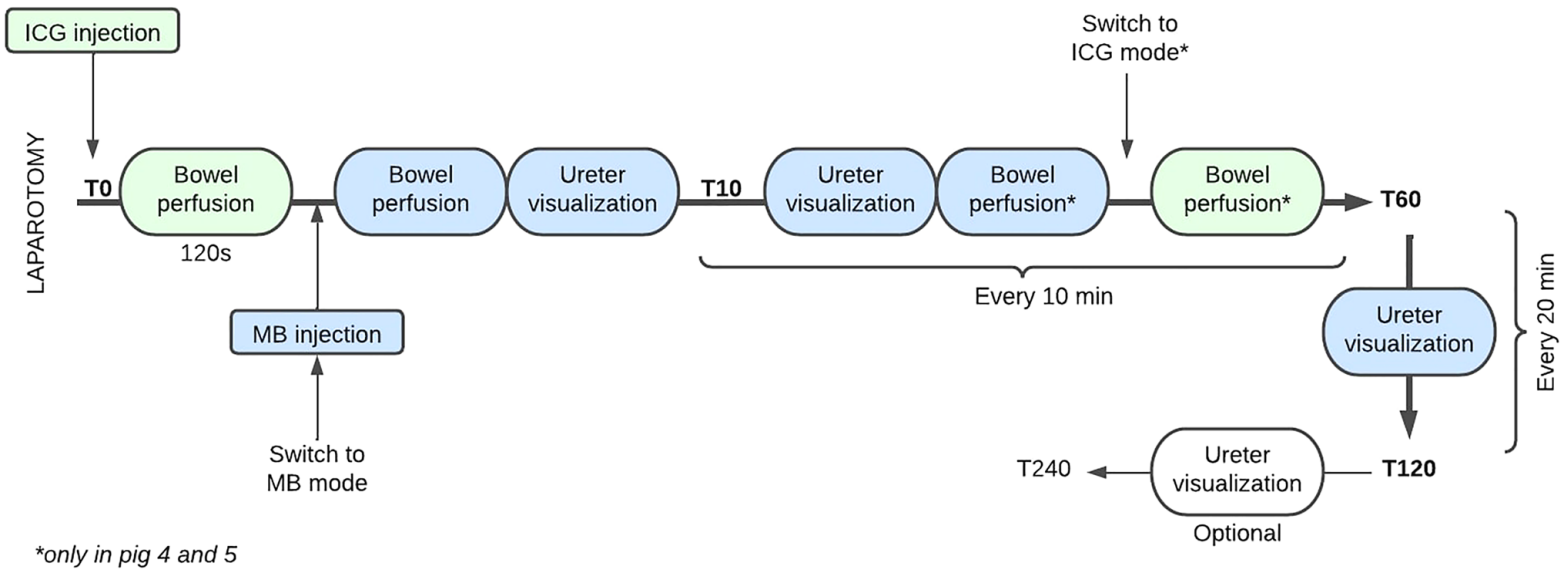
Overview of surgical procedure and measurements

After general anesthesia, a midline laparotomy was performed by an experienced surgeon. First, a loop of the small bowel with a length of approx. 15 cm, at 250 cm measured from the gastric pylorus, was selected as a region of interest. The camera system was switched to ICG mode followed by ICG injection. Bowel perfusion imaging was performed for at least 120 s. The same procedure was repeated under MB mode whereafter MB was administered. Consequently, the area where the left ureter would be expected was identified after 120 s and continuous left ureteral imaging in MB mode was performed until 5 min after dye administration. The latter was repeated every 10 min for a total of minimum 120 min (T120) after MB dye injection. Bowel perfusion imaging in both ICG and MB modes was performed in parallel for every 10 min in two pigs to investigate the washout pattern. In the other four pigs, only T0 measurements were taken. The identification of the right ureter occurred in the meantime, without any further recordings. An overview of the surgical procedure and measurements is shown in Fig. 1. At the end of the protocol, animals were euthanized with a lethal dose of pentobarbital (40 mg/kg).

### Statistical analysis and quantification of the fluorescence imaging

All NIRF videos were post-analyzed with Quest Spectrum software (ResearchTool v4.3 and TBR tool v1.0). The ureteral fluorescence imaging was assessed and quantified by calculating the fluorescence intensity (FI) and the target-to-background ratio (TBR; FI of target/FI background) [13, 17]. Background values were calculated approximately 1 cm on either side of the ureter, with solely retroperitoneal tissue. The FI of target was calculated based on the fluorescence signal during peristaltic contractions of the ureter by drawing a circle of interest in the corresponding region. Numerical variables were presented as means and standard deviation (SD) or median and interquartile range (IQR) where appropriate. To evaluate the statistical significance of numerical variable differences observed between groups and estimate group effect, a mixed model analysis was performed. Differences were considered significant when the *p* value was < 0.05. All the statistical analyses were performed with the SPSS^©^ software (version 27).

## Results

A total of six pigs were included in this study. Median weight was 39.25 ± 3.13 kg (IQR 36.00–42.25). All animals were followed for at least 120 min after dye administration. The maximum observation time took 420 min. Animal characteristics and clinical data are summarized in Table 1.

**Table 1:** Pig characteristics and clinical data

	Pig 1	Pig 2	Pig 3	Pig 4	Pig 5	Pig 6
Weight (kg)	41.00	43.00	42.00	37.50	36.00	36.00
MB dose (mg/kg)	0.75	0.75	0.50	0.50	0.25	0.25
*Total MB dose (mg)*	*30.75*	*32.25*	*21.00*	*18.75*	*9.00*	*9.00*
ICG dose (mg/kg)	0.20	0.20	0.20	0.20	0.20	0.20
*Total ICG dose (mg)*	*8.20*	*8.60*	*8.40*	*7.50*	*7.20*	*7.20*
Length of observation (min)	420	240	360	240	120	360
Number of ureters visualized *(n)*	2	2	2	1*	2	2

Italics values indicate a calculation of the row above (data of the MB or ICG dose)

^*^Due to renal agenesis, only right kidney in situ

### Ureter visualization analysis

**Fig. 2:**
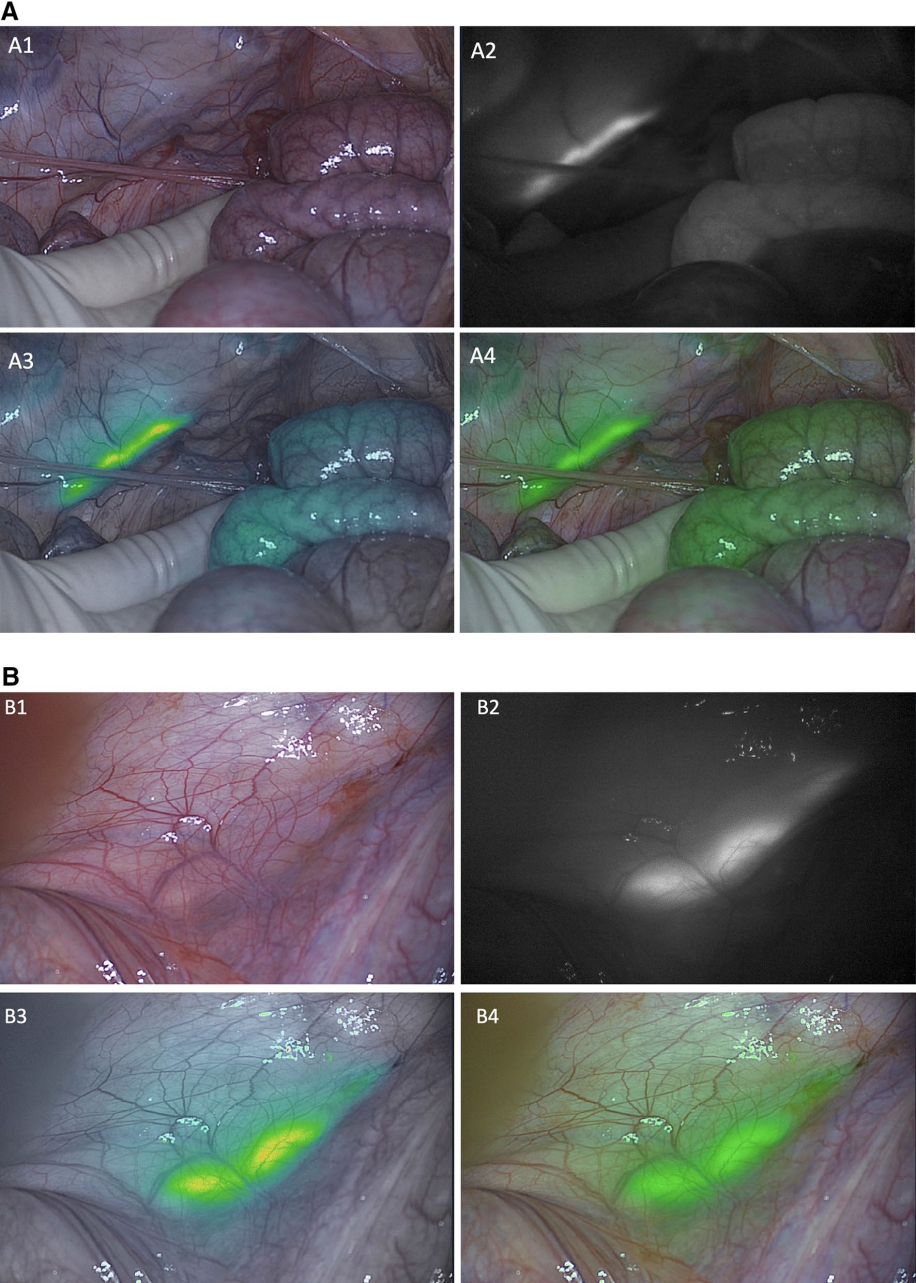
Examples of left ureter visualization with MB during peristaltic contraction. **A** (1) Color image of the surgical field, (2) NIRF image, (3) gradient overlay image, and (4) green overlay image of the left ureter in MB mode 10 min after MB injection (pig 2). **B** (1) Color image of the surgical field, (2) NIRF image, (3) gradient overlay image, and (4) green overlay image of the left ureter in MB mode 360 min after MB injection (pig 6) (Color figure online)

A total of 11 ureters in six pigs were identified. The reason why one ureter could not be identified was due to renal agenesis (only right kidney in situ). All ureters were clearly distinguishable from their surroundings. In all six experiments (based on three different doses of MB), the ureters were visible within five to ten minutes after dye administration and remained clearly visible until the end of every experiment (Fig. 2).

For the surgical team, no visual differences between the higher, middle, or lower doses were seen during the experiments. All evaluations showed a persistent clear delineation of the ureters in NIRF mode. The fluorescence signal was maximal during peristaltic contractions of the ureter. TBR values of all left ureters (except for pig no. 4, right ureter) at different time points are summarized in Table 2. The highest measured TBR was 8.89. All details about the mixed model analysis are presented in Table 3. Univariate tests did not show a significant difference when comparing the three dose groups (*p* = 0.345). The mean TBR value was the highest in group 2 (5.21 ± 0.37). The latter was confirmed in pairwise comparisons, which showed group 2 differed the most in mean TBR value compared to both other groups. The mean difference was 0.745 (*p* = 0.250) and 0.833 (*p* = 0.209) compared to group 1 and group 3, respectively. The relation between group and time was not significant (*p* = 0.855), indicating that the group effect did not significantly differ at different time points. As a consequence, the group effect was computed over all time points and showed non-significant effect either.

**Table 2:** TBR values for different time points

Pig (Ureter side and MB dose)	Pig 1	Pig 2	Pig 3	Pig 4	Pig 5	Pig 6
	Left	Left	Left	Right	Left	Left
	0.75 mg/kg	0.75 mg/kg	0.50 mg/kg	0.50 mg/kg	0.25 mg/kg	0.25 mg/kg
TBR 5	1.32 ± 0.12	1.18 ± 0.14	0.87 ± 0.25	0.96 ± 0.25	0.91 ± 0.23	2.98 ± 0.22
TBR 10	4.61 ± 0.35	5.70 ± 0.45	3.00 ± 0.29	5.61 ± 0.52	1.00 ± 0.16	5.89 ± 0.28
TBR 20	4.96 ± 0.41	3.95 ± 0.45	5.02 ± 0.37	7.88 ± 0.62	2.42 ± 0.28	5.98 ± 0.90
TBR 30	6.00 ± 0.15	4.08 ± 0.62	7.00 ± 0.34	5.35 ± 0.61	1.08 ± 0.43	4.76 ± 0.44
TBR 40	5.45 ± 0.31	3.51 ± 0.33	7.94 ± 0.35	4.34 ± 0.57	6.39 ± 0.44	–
TBR 50	4.29 ± 0.27	3.92 ± 0.36	5.75 ± 0.39	7.04 ± 0.59	4.47 ± 0.58	5.06 ± 0.26
TBR 60	4.46 ± 0.21	4.27 ± 0.50	6.42 ± 0.31	6.11 ± 0.51	6.41 ± 0.73	7.10 ± 0.57
TBR 80	6.58 ± 0.37	3.94 ± 0.41	8.89 ± 0.89	3.20 ± 0.38	3.69 ± 0.35	4.67 ± 0.33
TBR 100	3.97 ± 0.25	4.21 ± 0.49	4.63 ± 0.50	5.59 ± 0.46	7.51 ± 0.54	2.61 ± 0.31
TBR 120	7.02 ± 0.68	4.17 ± 0.50	3.86 ± 0.45	4.71 ± 0.34	8.84 ± 0.65	4.09 ± 0.32
TBR 180	–	2.60 ± 0.41	–	4.18 ± 0.37	–	–
TBR 240	5.51 ± 0.42	2.66 ± 0.24	5.46 ± 0.52	6.69 ± 0.68	–	–
TBR 360	–	–	3.86 ± 0.28	–	–	2.48 ± 0.20
TBR 420	4.36 ± 0.36	–	–	–	–	–

*TBR* target to background ratio

**Table 3:** Mean TBR values for different time points among different MB dose groups

*TBR time (min) *	Group 1 (0.75 mg/kg)	Group 2 (0.50 mg/kg)	Group 3 (0.25 mg/kg)	*p* value*
TBR 5	1.95 ± 1.46	0.92 ± 0.06	1.21 ± 0.04	0.826
TBR 10	3.45 ± 3.46	4.31 ± 1.84	5.16 ± 0.77	0.617
TBR 20	4.20 ± 2.52	6.45 ± 2.02	4.46 ± 0.71	0.374
TBR 30	4.76 ± 2.92	6.18 ± 1.17	5.04 ± 1.36	0.178
TBR 40	6.39 ± N/A	6.14 ± 2.55	4.48 ± 1.37	0.544
TBR 50	4.66 ± 0.42	6.40 ± 0.19	4.11 ± 0.26	0.405
TBR 60	6.76 ± 0.49	6.27 ± 0.22	4.37 ± 0.13	0.356
TBR 80	4.77 ± 0.14	6.04 ± 4.02	5.46 ± 1.87	0.760
TBR 100	5.00 ± 3.38	5.11 ± 0.67	4.09 ± 0.17	0.812
TBR 120	5.00 ± 1.28	4.29 ± 0.60	5.60 ± 2.02	0.751
Mean (95% CI)	4.46 ± 0.376 (3.31–5.62)	5.21 ± 0.37 (4.01–6.40)	4.38 ± 0.37 (3.18–5.57)	0.345

*TBR* Target to Background ratio

*Univariate tests within mixed model analysis

No adverse reactions were observed in any of the animals after MB administration. In the first pig, the left ureter was purposely transected at the end of the experiment. The leakage of urine due to ureteral damage could be clearly visualized with the NIRF imaging system (Supplementary, Figure A).

### Bowel perfusion analysis

**Fig. 3:**
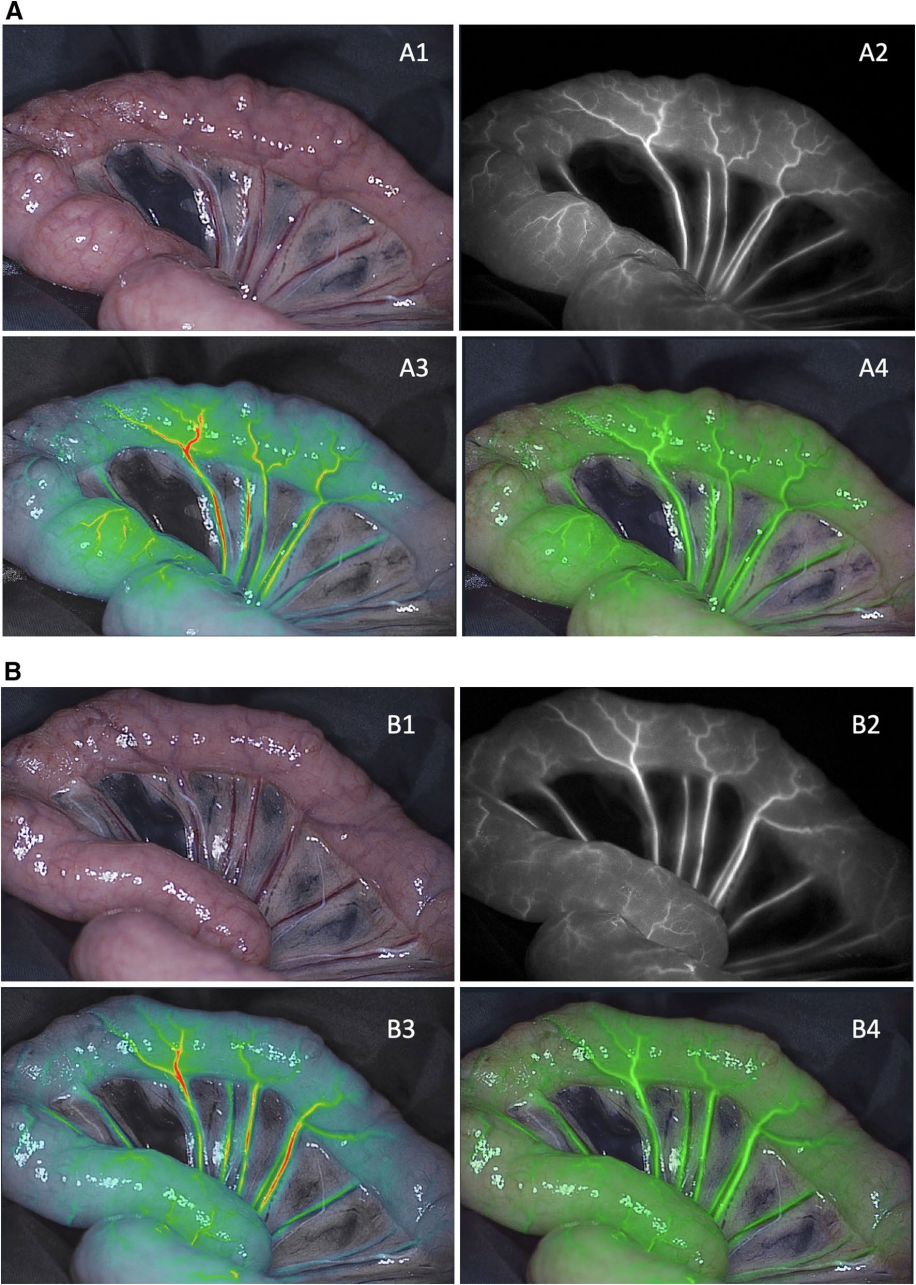
Bowel perfusion assessment in ICG and MB mode directly after dye injection (pig 4). **A** (1) Color image of the surgical field, (2) NIRF image, (3) gradient overlay image, and (4) green overlay image in ICG mode straight after ICG injection. **B** (1) Color image of the surgical field, (2) NIRF image, (3) gradient overlay image, and (4) green overlay image in MB mode immediately after MB injection (dose of 0.5 mg/kg) (Color figure online)

**Fig. 4:**
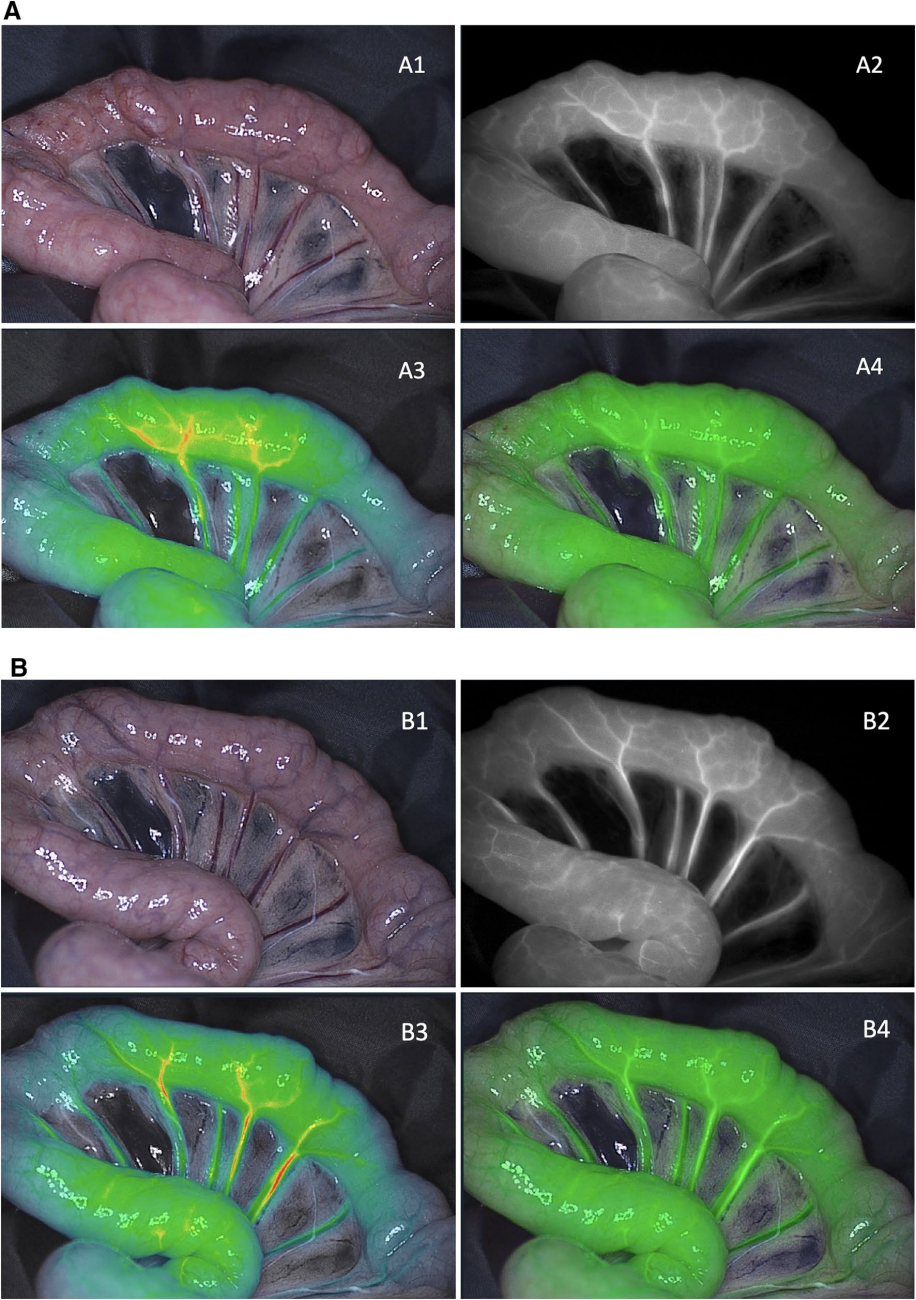
Bowel perfusion assessment in ICG and MB mode 20 s after dye injection (pig 4). **A** (1) Color image of the surgical field, (2) NIRF image, (3) gradient overlay image, and (4) green overlay image in ICG mode 20 s after ICG injection. **B** (1) Color image of the surgical field, (2) NIRF image, (3) gradient overlay image, and (4) green overlay image in MB mode 20 s after MB injection (dose of 0.5 mg/kg) (Color figure online)

**Fig. 5:**
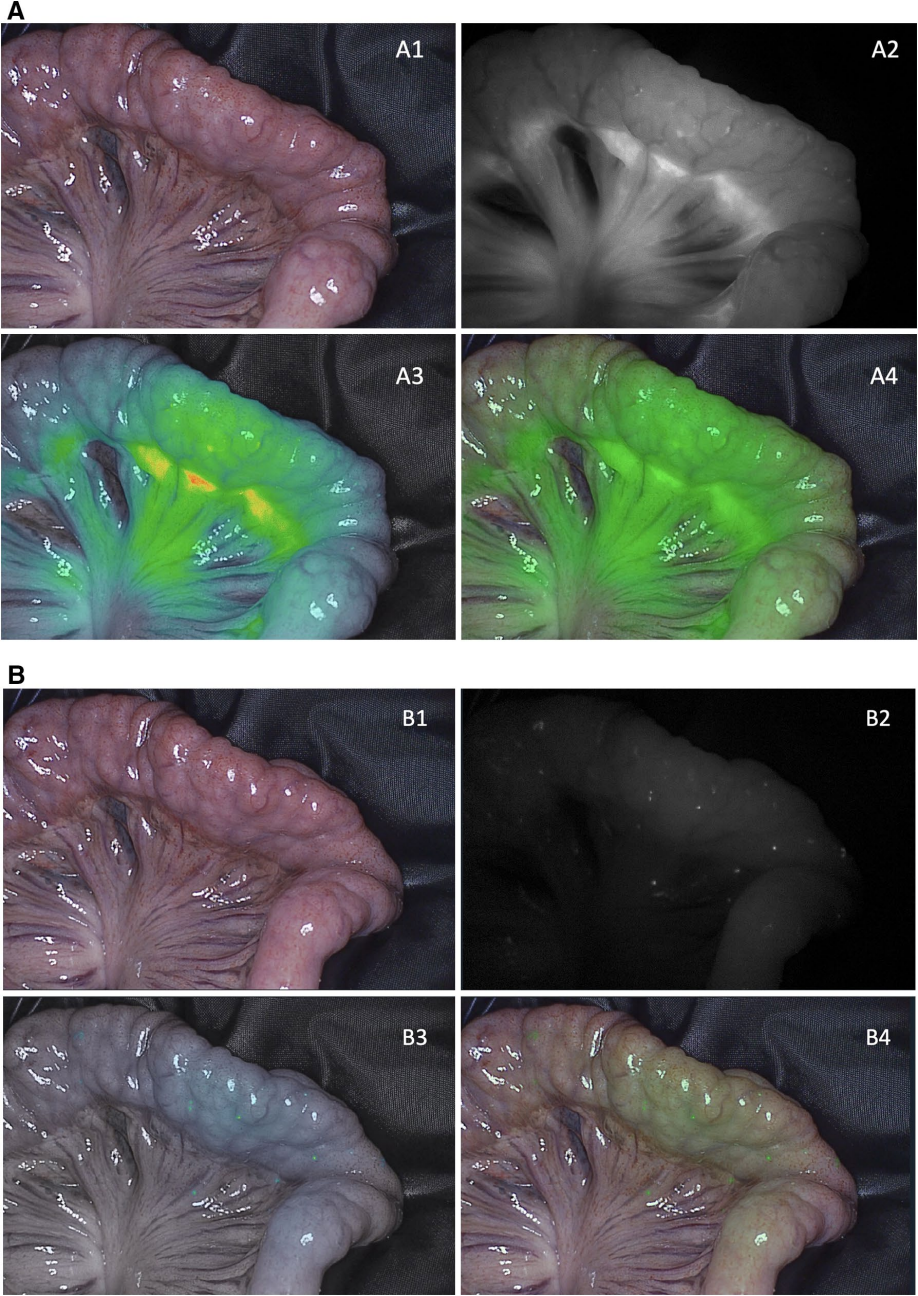
Bowel perfusion assessment in ICG and MB mode 50 min after dye injection (pig 4). **A** (1) Color image of the surgical field, (2) NIRF image, (3) gradient overlay image, and (4) green overlay image in ICG mode 50 min after ICG injection. **B** (1) Color image of the surgical field, (2) NIRF image, (3) gradient overlay image, and (4) green overlay image in MB mode 50 min after MB injection (dose of 0.5 mg/kg) (Color figure online)

In all pigs, a clear macroscopic NIRF visualization of the perfusion in ICG and MB mode was achieved in all pigs within a few seconds after dye administration (Fig. 3). After 20 s, maximal intensities were reached (Fig. 4). No interference between ICG and MB was observed when switching to either mode. In pigs 4 and 5, bowel perfusion was assessed every 10 min for at least 60 min to investigate washout and fluorescence intensities over time. After 50 min of dye administration, ICG was still clearly visible while MB was almost no longer visible in pig 4 (MB dose of 0.5 mg/kg) (Fig. 5). After 60 min, only a few spots of MB dye were still visible. Pig 5 (MB dose of 0.25 mg/kg) also showed an earlier washout time of MB compared to ICG, after 40 min of MB injection. No adverse reactions were observed in any of the animals after ICG administration.

## Discussion

This study demonstrated that simultaneous ureteral and bowel perfusion imaging in the same surgical procedure using a single commercially available NIRF imaging system and with the use of FDA-approved fluorescent dyes is feasible. The current findings provide surgeons with a potentially powerful tool to enhance the visibility of the ureter and assess bowel perfusion during colorectal procedures.

With MB, clear identification of the ureters was achieved under NIRF imaging in all animals, as well as assessment of intestinal perfusion. The pigs were allocated to 3 different dose groups to determine the optimal dose of MB. Although no significant differences in TBR values were found among groups, the dose of 0.50 mg/kg MB appeared to be the most optimal with the highest TBR values in this animal study. A previously described optimal dose in the first human study using MB to identify the ureter was 0.25 mg/kg [18]. Additionally, we found the ideal time to administer MB to be 10 min prior to requiring ureter delineation and that ureteral imaging remains possible at least until 240 min after a single bolus of MB dye administration.

Bowel perfusion assessment was successful in all pigs with ICG. A key finding is that we have demonstrated that bowel perfusion can be visualized with MB as well. Cwalinski et al. created an overview of the role of MB as a fluorophore in a surgical setting [19]; however, bowel perfusion assessment was not mentioned. The latter suggests that MB may represent a versatile substitute for ICG in intestinal perfusion imaging, especially in cases where there is also a need for intraoperative ureteral identification or when ICG is contraindicated. Another clinically relevant finding of this study is that there was no interference between ICG and MB in bowel perfusion assessment. As a result, MB alone may enable us to clearly assess intestinal perfusion in combination with ureteral imaging without the need for ICG.

In several studies [5, 17, 20], our groups have thoroughly explored the potential of novel dyes for the purpose of intraoperative ureteral imaging. Although showing promising results, such novel dyes are still in an experimental phase and it is expected that it will take several years before they will be available for clinical use. A previous pre-clinical study by our group has demonstrated the simultaneous assessment of bowel perfusion and ureteral delineation with a single dye [12]. However, the dye used in that study (IRDye® 800BK) is not yet approved for clinical use. In contrast, MB has been widely used in humans with a good safety profile. It is cheap and clinically available. In addition, MB is approved by the US Food and Drug Administration (FDA) for many indications [21].

We believe that the use of a single dye for bowel perfusion assessment and ureteral imaging has several advantages. Most importantly, it reduces the potential risk of adverse reactions as only one dye is administered and contributes to the efficiency of the procedure. One point of attention is the fact that MB can only be used in patients with adequate renal function and the intensity of the ureteral signal is influenced by the peristaltic movement of urine through the ureter [18]. There are some known adverse effects after MB administration such as hypertension, dyspnea, hemolysis, methemoglobinemia, nausea and vomiting, and pain in the chest when administering doses above 2–7 mg/kg. Refractory hypotension and skin discoloration are known upon administration of 20–80 mg/kg [21]. As the previous mentioned doses are much higher than needed for ureter delineation and bowel perfusion assessment as demonstrated in this study (even visible with the lowest dose of 0.25 mg/kg), such adverse events are not expected for this indication. MB is currently safely used for visualization of thyroid and parathyroid glands, pancreatic neuroendocrine tumors, and breast cancer tumors and sentinel nodes within therapeutic doses of < 2 mg/kg [19].

The current study also demonstrated a faster washout of MB compared to ICG during bowel perfusion assessment, which is known to remain fluorescent for long periods after dye administration. This finding may allow for repeated MB bolus administrations within the same procedure for the purpose of perfusion assessment. We hypothesize that three MB dye characteristics could play a role in the faster washout. First, the molecular weight of both dyes is different, which may result in a difference of diffusion of the dyes into the capillaries; MB has a molecular weight of 319.85 Da as compared to 774.963 Da for ICG [22]. Secondly, MB is more hydrophobic than ICG, which has two hydrophobic and two hydrophilic molecule groups [23–25]. Thirdly, the binding properties of both dyes are probably different: ICG tends to bind to plasma proteins [26], whilst this is not well described for MB. An earlier MB washout time can be beneficial, as a repeated dye injection for bowel perfusion assessment may be necessary during a surgical procedure. When MB is completely washed out, a second dose can be given without the interference of previous signals. We also observed a difference between the washout time of an MB dose of 0.5 and 0.25 mg/kg (50 and 40 min respectively). The differences observed in both pigs are probably due to the MB dose administered.

A recent clinical pilot study successfully demonstrated that it is feasible to delineate the ureters with MB and assess the perfusion with ICG using the same camera system [27]. The authors included 12 patients who underwent complex open or laparoscopic colorectal surgeries and demonstrated successful ureteral delineation with MB in 91.6% of cases, and successful bowel perfusion assessment with ICG in all cases. In this pilot study, all measurements were only taken immediately after dye injection, without a follow-up in time. Besides, bowel perfusion was not visualized with MB. We believe, as demonstrated in our study, that the next step would be to focus only on MB fluorescence imaging for ureteral and perfusion imaging. The relatively fast washout of the MB dye may allow for repeated MB administration; one prior to the surgical procedure for ureteral imaging and one during the procedure for perfusion imaging. Based on the results of this study and previous articles in the literature, we have designed a further animal study in which intestinal perfusion quantification for MB compared to ICG will be explored in more detail.

One of the main limitations of this animal study is the small sample size. However, for the feasibility of our hypothesis and to respect the 3R principle (replace, reduce, refine) in animal research as described by Russell and Burch [28], the current number of animals was deemed sufficient. The current results must be interpreted with caution and human studies are necessary to evaluate the reproducibility of our finding in a clinical setting. In addition, while most of the elective abdominal clinical procedures are performed laparoscopically, in this study due to logistical reasons the camera system used was an open camera system (for use after laparotomy). Fortunately, a laparoscopic variant of the camera used in this study is commercially available.

The duration of the study per animal was not exactly the same. The study setup was to have a follow-up time of 120 min (most related to a clinical setting) of observation in all animals. This requirement was met in all animals. As an interesting additional finding, we were also interested in the fluorescence signal of the ureter over time. The difference in the timings in the various animals after 120 min can be simply explained due to logistical reasons. As we performed more than one operation per day, the timing of the start of the procedure was the most important factor in the maximum time of follow-up after dye injection.

This feasibility animal study has provided the basis for further, larger human studies evaluating dual-imaging camera systems, using only one single dye (MB) for ureteral imaging and bowel perfusion assessment. In line with ongoing animal intestinal perfusion quantification research by our team, we believe a next step should be to further investigate the use of MB in assessing bowel perfusion, as this is not well described [19] and take this research to the human setting in colectomy procedures.

## Conclusions

Our study shows the feasibility of simultaneous fluorescence angiography with ICG and ureteral imaging with MB in the same surgical procedure, using the same commercially available NIRF imaging equipment. As both dyes can be used in humans, we believe that there is high potential for clinical translational. Additionally, this dual camera system allows for the simultaneous assessment of bowel perfusion and ureteral visualization, using a single dye (MB). Further human studies are necessary to translate our findings to clinical application.

## Supplementary Information

Below is the link to the electronic supplementary material.

Supplementary file1 (DOCX 3671 KB)
