# Supplementary material for: Simultaneous fluorescence imaging of bowel perfusion and ureter delineation using methylene blue: a demonstration in a porcine model
Source: Surg Endosc. 2023 May 30;37(9):6779–90. doi: 10.1007/s00464-023-10142-6 (PMC10462514; doi:10.1007/s00464-023-10142-6)
Supplement: Supplementary file 1 — Supplementary file1 (DOCX 3671 KB) [file 464_2023_10142_MOESM1_ESM.docx]

**Supplementary**

Figure A. Left transected ureter visualization in pig 1 with MB.

**
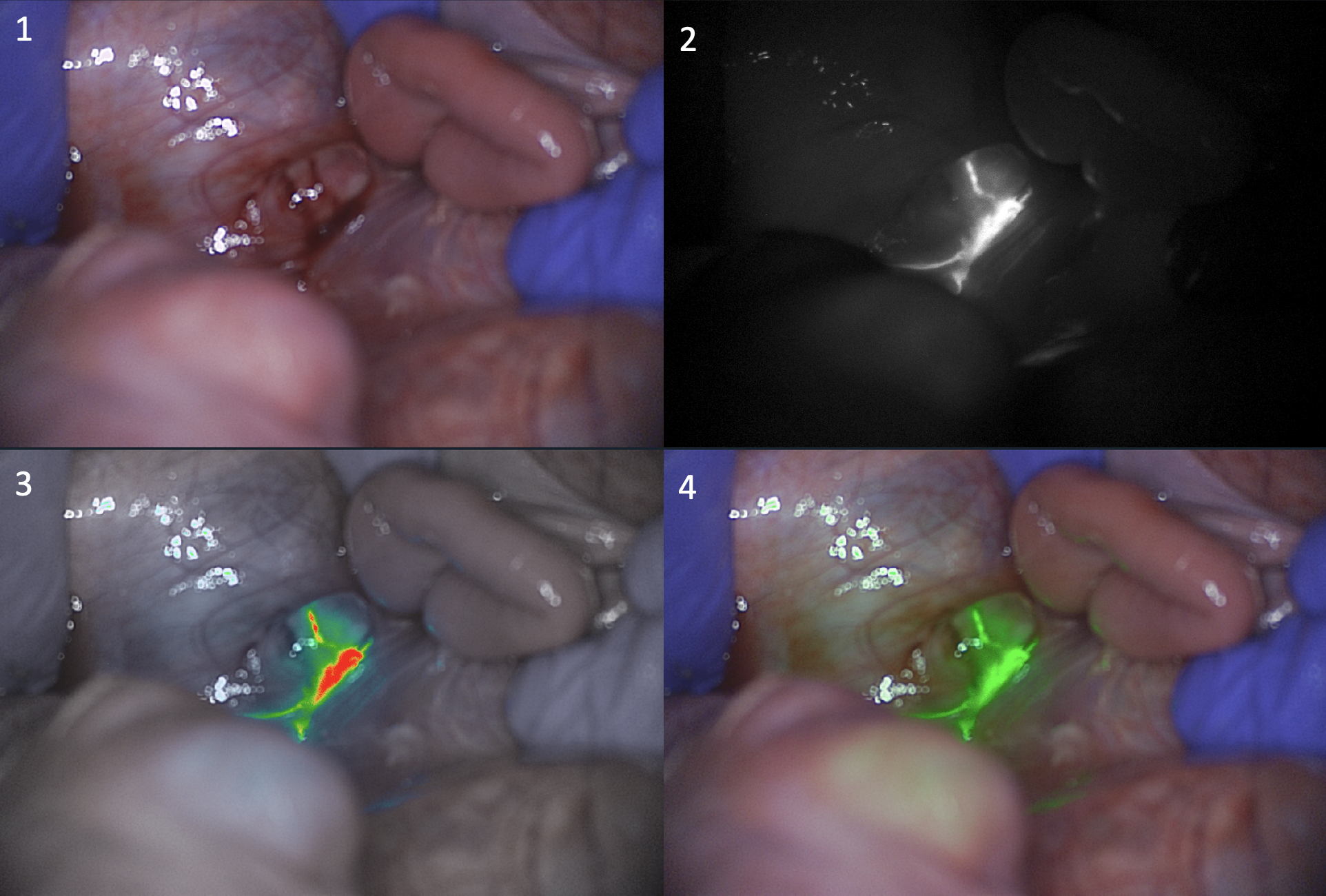
**

(1) Color image of the surgical field, (2) NIRF image, (3) gradient overlay image, and (4) green overlay image in MB mode 7 hours after MB injection, after ureteral transection.
